# Exercise as a Promising Agent against Cancer: Evaluating Its Anti-Cancer Molecular Mechanisms

**DOI:** 10.3390/cancers15215135

**Published:** 2023-10-25

**Authors:** Maria Spanoudaki, Constantinos Giaginis, Dimitra Karafyllaki, Konstantinos Papadopoulos, Evangelos Solovos, Georgios Antasouras, Georgios Sfikas, Athanasios N. Papadopoulos, Sousana K. Papadopoulou

**Affiliations:** 1 Department of Nutritional Sciences and Dietetics, School of Health Sciences, International Hellenic University, 57400 Thessaloniki, Greece; maryspan1@gmail.com (M.S.); papadnas@ihu.gr (A.N.P.); souzpapa@gmail.com (S.K.P.); 2 424 General Military Hospital of Thessaloniki, 54621 Thessaloniki, Greece; k.a.papadopoulos@army.gr (K.P.); geosfik@gmail.com (G.S.); 3 Department of Food Science and Nutrition, School of Environment, University of Aegean, 81400 Lemnos, Greece; g.antasouras@gmail.com; 4 Department of Nutrition and Dietetics, School of Physical Education, Sport Science and Dietetics, University of Thessaly, 42132 Trikala, Greece; dkarafyllaki@uth.gr

**Keywords:** exercise, cancer, molecular mechanisms, prevention, inflammation, hormones, oxidative stress, immune system, personalized strategies, co-treatment

## Abstract

**Simple Summary:**

Cancer is the main cause of mortality and morbidity worldwide. The incidence of physical inactivity is gradually increasing, reaching alarmingly high levels. Several review articles have summarized the potential beneficial effects of exercise in several aspects of the lifestyles of patients with cancer. However, only a few outdated review articles have focused on the anti-cancer molecular mechanisms of exercise. In view of the above considerations, the aim of this review study is to highlight the interrelationship between cancer and exercise on a molecular basis, evaluating the most well-established molecular mechanisms of exercise against cancer development and progression. This review study aims to provide convincing evidence that exercise may be a complementary part of the medical treatment of cancer patients, underlining the most important anti-cancer molecular mechanisms, which may contribute to cancer prevention and co-treatment.

**Abstract:**

Background: Cancer cases are continuously increasing, while the prevalence rates of physical inactivity are also continuously increasing. Physical inactivity is a causative factor in non-communicable diseases, including cancer. However, the potential beneficial effects of exercise on cancer treatment have not received much attention so far. The aim of this study was to highlight the relationship between cancer and exercise on a molecular basis. Methods: Comprehensive and in-depth research was conducted in the most accurate scientific databases by using relevant and effective keywords. Results: The mechanisms by which exercise may reduce cancer risk and/or progression may include the metabolic profile of hormones, systemic inflammation reduction, insulin sensitivity increase, antioxidant capacity augmentation, the boost to the immune system, and the direct effect on the tumor. There is currently substantial evidence that the effect of exercise may predict a stronger association with cancer and could supplementarily be embedded in cancer clinical practice to improve disease progression and prognosis. Conclusion: The field of this study requires interconnecting the overall knowledge of exercise physiology with cancer biology and cancer clinical oncology to provide the basis for personalized targeting strategies that can be merged with training as a component of a holistic co-treatment approach to optimize cancer healthcare.

## 1. Introduction

Scientific evolution has resulted in the holistic treatment of the oncology patient through rehabilitation programs. However, the implementation of therapeutic exercise is not widely established in a daily clinical practice. Although randomized clinical trials have significantly demonstrated the anti-cancer protection of exercise, deepening scientific knowledge, the results are sometimes contradictory. The heterogeneity of tumor malignancies and the individualized characteristics of each population or even the personalized features of each patient are usually the cause of this inconsistency [1].

Cancer is one of the main causes of mortality in 57 countries worldwide and is characterized by a quick and uncontrolled proliferation of cells that do not follow the control mechanisms of cell division and may be sited on an organ or be metastatic [2]. Characteristically, in 2012, 14,106 new cancer cases and 82,106 deaths were diagnosed in the United States of America, while in the next two decades, the rate of new cases is expected to highly increase by 70% [3]. The latest global estimations have revealed that 1.4 billion adults (one third of the world’s adult population) do not meet the recommended level of physical activity to improve and protect their health. Alarmingly enough, this issue has remained highly unchanged, and it has further been adopted during the pandemic period due to the COVID-19 pandemic [4,5]. The distribution of cancer types shows an increasing trend over the last few years. According to World Health Organization (WHO) statistics, the most common new cases of cancer in 2020 were breast, lung, colon, prostate, cervical, skin, and stomach cancer [2,3,4].

Levels of physical inactivity in high-income countries (36.8%) were more than two-fold higher compared to low-income countries (16.2%) among adults in 2016. In most countries women still are more inactive than men, particularly in the Eastern Mediterranean and the United States of America regions [6]. On the other hand, cancer disease has been attributed to the interactions of genetic and environmental factors. Data from both laboratory and observational research have suggested that modifiable risk factors, such as physical inactivity, smoking, dietary habits, and alcohol consumption, can influence the risk of cancer recurrence and overall survival after diagnosis [7].

Globally, one third of adults are not physically active. Data from WHO surveys have revealed that 64% of adults are sedentary for more than four hours a day. Sedentary behavior has been considered as a major factor in the burden of non-communicable diseases, including cancer. A burden of 10% for breast cancer and 10% for colon cancer has been reported to be related with sedentary behavior [7,8].

A plethora of epidemiological, retrospective, prospective, and case control studies has described the potential benefits of physical activity/exercise in relation to cancer risk [8], revealing that regular exercise may promote protection against specific types of cancer, such as breast cancer in menopausal women, bowel (colon), and possibly prostate, endometrial, lung, and perhaps pancreatic cancer [9], reducing the overall incidence of these cancer types by a percentage of 40% [10]. Some evidence has also shown that the relative risk is increased in colon (RR = 2.0) and breast cancer (RR = 1.5) in people with sedentary lifestyles, whereas a 75% risk reduction for breast cancer and 22% for colorectal cancer in people with increased physical activity levels has been reported [2,11]. The Center for Disease Control and Prevention (CDC) reports also that physically active individuals have a lower risk of developing breast, colon, lung, and endometrial cancer [12].

Most studies support that exercise is generally safe for people being treated for cancer and has a positive effect on reducing the risk of cancer development and progression in specific organs [11,12]. The way in which exercise may prevent the development of cancer is still under investigation. Physical activity seems to considerably improve physical performance and muscle strength, also promoting several aspects of patients’ mental health. Exercise can reduce tumor development through multiple mechanisms such as the following: (a) vascularization and perfusion, (b) immune function, (c) tumor metabolism, and (d) muscle–cancer interaction [13]. Evidence of these mechanisms is currently emerging, but more intervention studies are still recommended to establish the cause–effect relationship between these mechanisms and the control of tumor genesis and growth [13].

Notably, there are some review articles that have summarized the potential beneficial effects of exercise on quality of life, depression, cancer-related cognitive impairment, functionality status, chronic pain, cancer-related fatigue, radiotherapy-related dysphagia, psycho-emotional status, cancer cachexia, sarcopenia, chemotherapy toxicity, bone health, and sleep disturbances [14,15,16,17,18,19,20,21,22,23,24,25,26,27]. However, only a few outdated review articles have focused on the anti-cancer molecular mechanisms of exercise. In this aspect, the aim of this study was to critically highlight and scrutinize the relationship between cancer and exercise on a molecular basis, convincing the scientific world to promote exercise as an integral part of the medical treatment of cancer patients.

## 2. Materials and Methods

Comprehensive and in-depth research was conducted in the most accurate scientific databases, e.g., PubMED, Cochrane, Elsevier, Scopus, and Google Scholar, by using relevant terms: cancer, tumor, malignancies, exercise, cancer progression, cancer survival, cancer treatment, exercise, physical inactivity, anti-cancer molecular mechanisms, hormones, inflammation oxidative stress, cancer type, proliferation, mutation, etc. In vitro and in vitro animal studies were excluded from the analysis. Only studies in humans were included. Peer reviewed journal papers were included only if they were written in English.

The research was extended with the searching of reference lists of related reviews, and hand-searching key journals, commentaries, editorials, and abstracts in congressional proceedings. The recovered studies were further and thoroughly examined for related studies included in their manuscripts. To enhance consistency among reviewers, all reviewers examined all the recovered articles, discussed their findings, and modified the screening and data extraction manual before the initiation screening for this review. All reviewers co-operating in pairs sequentially assessed the titles, abstracts, and the full text of all articles recognized by our research for possibly related articles.

**Figure 1:**
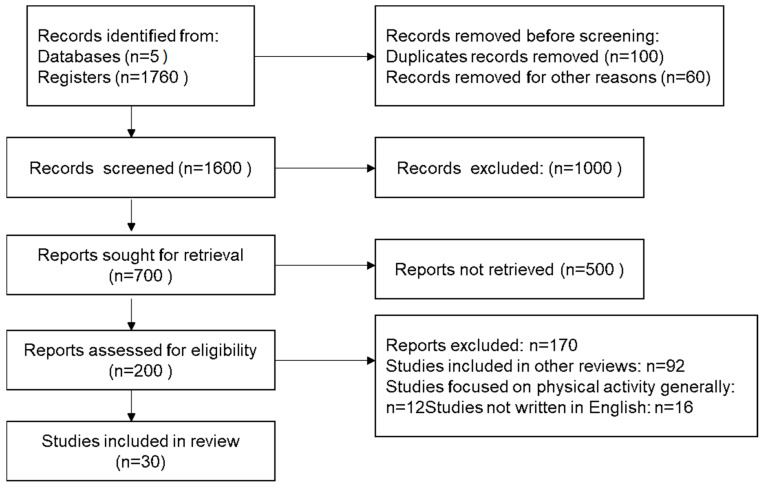
Flow chart diagram for the studies included in the present review.

Any reviewers’ disagreement on article selection and data extraction was solved by consensus and analysis with all the authors/reviewers if it was necessary. Inclusion criteria were any retrospective, prospective, cross-sectional, descriptive, and preliminary/pilot clinical and Randomized Control studies (RCTs). The findings were taken into consideration according to relevance and the most relevant ones were selected based on the inclusion and exclusion criteria reported above (Figure 1).

## 3. Results

### 3.1. Exercise and Its Molecular Mechanisms against Cancer

**Figure 2:**
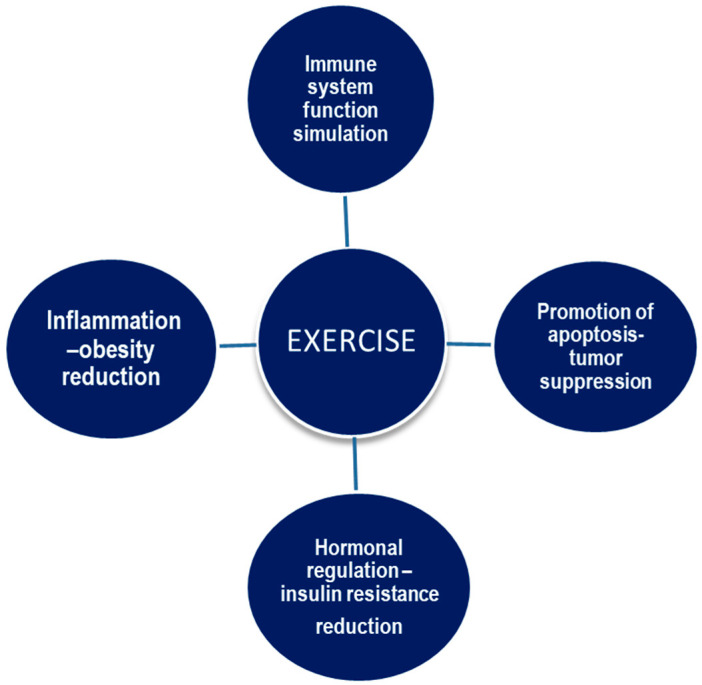
The main effects of exercise on cancer development and progression.

It is well documented that tumor growth is mediated by complex and multidimensional interactions between the systemic environment, the tumor cells, and the tumor microenvironment. The tumor microenvironment is directly affected and responds to several secreted growth factors, hormones, angiogenic factors, cytokines, and many other cell types, including immune system cells, that make up the systemic environment [28,29]. Elevated circulating levels of certain inflammatory cytokines and angiogenic factors such as tumor necrosis factor-α (TNFα) and interleukin-6 (IL-6), as well as metabolic growth hormones (leptin and insulin), have been associated with a higher risk of cancer recurrence and mortality in several solid tumors. One related pathway that may also play a key role in providing support for the anti-cancer benefits of exercise is the inflammation–immune system axis [29,30]. The main effects of exercise on cancer development and progression are depicted in Figure 2.

More to the point, exercise modulates the immune system by affecting cytoxins, growth factors, metabolites, angiogenesis, and other factors [31]. NK cells are the most sensitive cells of the immune system to exercise, and they exhibit acute mobilization and turnover during exercise. These cells have recently been found to exert a crucial role in anti-cancer protection, and exercise seems to be the key mediating factor in this relationship [32]. Generally, peak NK cell recruitment is reached within 30 min of endurance exercise and levels remain elevated for up to three hours, while prolonged endurance exercise (half marathon) leads to an elevation of histone acetylation and natural killer group 2D (NKG2D) expression for a minimum of 24 h post-running [33]. Both histone acetylation and NKG2D expression, a functional NK cell marker, are elevated for at least 24 h after the run.

The above elevation is ascribed to catecholamines (epinephrine, norepinephrine), which are also elevated during moderate- to high-intensity exercise and appear to drive NK cells into circulation. During exercise, myokines and peptides are released into circulation, and peptides drawn from muscle fibers are also released into circulation [34]. Myokines appear to drive energy sources to exercising muscles by modulating their metabolic responses during training cessation. They also influence the function and activity of NK cells, creating an axis that links the exercise to their setting and may have anti-cancer effects and promotional functions depending on their type and environment [35]. Myokines such as oncostatin M (OSM), secreted protein acidic rich in cysteine (SPARC), irisin, and decorin can directly restrain tumor development by blocking cell proliferation and migration and promoting tumor cell apoptosis. SPARC has been found to be released from skeletal muscle into circulation after a single period of exercise in healthy humans and in rats with colorectal cancer. Furthermore, myokine production by skeletal muscles may be involved in the protective role of exercise [36].

It should be noted that exercise-induced myokines, including IL-6, IL-8, IL-15, brain-derived neurotrophic factor (BDNF), and leukemia inhibitory factor, are released from muscle fibers. Insulin sensitivity is also elevated by these cytokines, while the production of pro-inflammatory cytokines is decreased [37]. Exercise also controls blood flow and oxygen consumption, affecting NK cell function [30]. Endurance exercise has also been associated with increased vascularization, angiogenesis, and core body temperature and therefore may affect circulation and the ability of NK cells to regulate and control tumor growth, while other studies have shown a decrease in angiogenesis and perfusion within the tumor [12]. On the other hand, the intensity of an exercise program is related to the optimal (or not) result as moderate-intensity exercise improves immune function, but high-intensity exercise can lead to its suppression [9]. Exercise also regulates the cellular immune system by immobilizing immune cytotoxic cells in circulation through shear stress induced by blood flow and adrenergic signaling. These activated immune checkpoint cytotoxic cells search the body to identify and destroy the mutant cells [38].

### 3.2. Sex Steroid Hormones and Blood Serum Hormone Levels

As far as healthy premenopausal women are concerned, exercise has been related with decreased estrogen and progesterone amounts [31]. One suggested mechanism for decreasing the probability of breast cancer by exercise concerns the impact on steroid hormones and menstrual cycle function in women. In female athletes, there is a delay in menarche and therefore a reduction in exposure to estrogenic factors [9,39]. Early menarche implies increased estrogen levels during reproductive age and steroid hormones are positively associated with an increased risk of breast cancer [40]. Estrogen and progesterone are mitogenic factors for the breast and their reduction in blood serum is associated with a reduction in the ability of cells to multiply. The shortening of the luteal phase observed in response to exercise may also lead to a decrease in cell multiplication [11,31]. However, the latest evidence supports that endurance training in premenopausal women has little effect on sex steroid hormones.

In postmenopausal women, an increase in physical activity has been associated with low serum levels of estradiol, estrone, and androgens, especially after adjustment for body mass index (BMI). The reduction of body fat in active women can lead to a reduction in 16-Hydroestrone (C-16a) with estrogenic activity [30]. Interesting is the observation of the process of aromatization of androgens to estrogens. Exercise affects estrogenic metabolism [31,41]. Notably, in female athletes increased levels of C-2-hydroxylated estrogens (without estrogenic action) and decreased hydroxylation products were found. The increase in 2C-hydroxylated metabolites has positively been associated with a reduction in the risk of prostate cancer [40,42].

Chronic endurance exercise in men may affect the serum levels of sex hormones [43]. In fact, prostate tumors appear to respond to androgens. More to the point, some studies have demonstrated that endurance exercise may reduce androgen levels either by modifying them or by limiting their availability (increasing their binding protein levels). However, other studies did not support a relationship between exercise and cancer risk by androgen level reduction and concluded that exercise may be positively associated with prostate cancer incidence in men with increased muscle mass. Intense aerobic exercise in young men and adolescents has also been shown to lead to an acute increase in androgens after it ends and a return of testosterone to baseline levels [29,31].

About 50% of Leydig cells are removed during unilateral orchectomy and irregular testosterone levels have been found in survivors of testicular cancer. In healthy subjects, it has been shown that testosterone suppression can moderate the responsiveness to resistance training. It is currently not known whether chemotherapy patients have a reduced response to exercise due to treatment-induced hypogonadism [44]. Notably, no effect has been found of resistance training on plasma cytokine concentrations in testicular cancer patients compared to the inactive ones [45].

### 3.3. Oxidative Stress/Free Radicals/DNA Damage and Repair

Exercise puts the body under oxidative stress and increases oxygen free radicals. However, the body’s antioxidant defense increases despite claims otherwise. Thus, exercise can become beneficial or not, depending on the intensity and the load of the exercise program. Exercise has been shown to increase the activity of antioxidant enzymes (superoxide dismutase, catalase, and glutathione peroxidase) [34]. Research findings are contradictory, where there are arguments that aerobic and prolonged exercise can lead to the depletion of defense antioxidant mechanisms, resulting in DNA damage and increased cell proliferation. The association between DNA damage and exercise can be partly ascribed to the hostage theory, which is rather one-dimensional and therefore limited [46]. However, such a case has not been reported yet. The body’s antioxidant capacity declines with age, but moderate exercise in elderly populations may mitigate this reduction [47].

Furthermore, exercise, particularly intense exercise, produces reactive oxidant species (ROS), which can cause oxidative stress on DNA if they are elevated, and could potentially lead to the development and growth of cancer. In response to this transient increase in ROS, especially after regular exercise, an adaptive regulation of antioxidant genes occurs, resulting in greater synthesis of antioxidant enzymes such as catalase, glutathione, and superoxide dismutase [48]. Moreover, submaximal exercise activates the expression of the nuclear factor erythroid 2-related factor 2 (Nrf-2) in normal prostate tissue compared with men that are less physically active. The Nrf-2 protein stimulates the generation of antioxidant enzymes and activates other genes with a protective role [44].

Substantial research has confirmed that trained individuals also have higher levels of antioxidant enzymes, which could potentially increase their defense against environ- mental and ingested oxidative carcinogens. If nutritional deficiencies exist that reduce antioxidant enzyme production or if intensive physical activity is performed by older adults, where this adaptation procedure is known to be a slower process, there is a risk that intense exercise may be harmful for them. Thus, it is essential to provide the consumption of foods containing ingredients rich in polyphenols, which increase the regulation of antioxidant enzymes [49]. Finally, studies converge on the concept that moderate exercise can induce the expression of antioxidant enzymes that in turn express proteins with strong antioxidant activity, thus limiting cancer risk [50].

### 3.4. Insulin and the Insulin-like Growth Factor (IGF) Axis

The role of insulin in carcinogenesis in a variety of organs such as the pancreas, breast, colon, liver, and endometrium are of particular interest. In cancer patients (without diseases associated with hyperinsulinemia), serum insulin levels have been reported to be elevated. The prevalence of non-insulin-dependent diabetes has positively been associated with cancer incidence [51]. Insulin levels and the risk of non-insulin-dependent diabetes are strongly influenced by body fat distribution and physical activity. More to the point, exercise increases insulin sensitivity, decreases plasma insulin and C peptide, and increases glucagon levels. However, the effects of exercise on the insulin receptor, insulin kinase receptor, insulin substrate receptor, and its phosphorylation are modified in obese subjects and are still under investigation [28].

On the other hand, hyperinsulinemia causes an increase in insulin growth factor (IGF)-1 and a decrease in its binding proteins. Notably, IGF-1 exerts a primary role in carcinogenesis. Chronic exercise reduces insulin resistance even in individuals with impaired glucose tolerance, decreases IGF-1 levels, and increases its binding proteins [52]. The effect of exercise on increasing IGF-1 levels remains still unclear, as the results of the currently available studies are contradictory. Regarding its binding protein, IGBP-3, a small number of studies have shown an increase with higher levels of physical activity [9,53]. Most of the studies have documented that increasing physical activity did not affect IGF-1 levels, while the rest shows an increase in this factor. Regarding its binding protein, IGBP-3, a small number of those studies have shown an elevation with increasing levels of physical activity [52,54]. Recent research has revealed that exercise may significantly exhibit decreased post-operative IGF-1, CRP, TNFα, and Il-6 levels in adult and older adult women with breast cancer [52,55]. Duration of training appears to regulate this relation, which is ascribed to the duration of training with predominantly over 12 weeks of exercise that decreased the factors mentioned previously. On the other hand, a session of training period less or equal to 12 weeks lowered only CRP levels [37,56].

### 3.5. Inflammation

Systemic inflammation has been linked to several chronic diseases, including cancer. Chronic inflammation has been associated with several stages involved in carcinogenesis, including cell transformation, promotion, survival, proliferation, invasion, angiogenesis, and metastasis. Pro-inflammatory factors such as C reactive protein (CRP), amyloid-α, IL-6, and TNF-α, and anti-inflammatory factors such as adiponectin, are being investigated as markers of increased risk and disease prognosis [56]. The chronic inflammatory microenvironment is mainly derived from macrophages. These macrophages, along with other leukocytes, produce high levels of reactive oxygen and nitrogen species to fight inflammation [57]. However, in an environment of continuous tissue damage and cellular proliferation, the survival of these infection-fighting factors is harmful. Mutagenic factors, such as peroxynitrite, which react with DNA and cause mutations in proliferating epithelial and stromal cells, can be produced [58]. Macrophages and T-lymphocytes may release TNF-α and macrophage migration inhibitory factor to exacerbate DNA damage [30,58]. Training appears to reduce systemic inflammation independently or in combination with weight control or body composition. In this context, several cross-sectional studies have supported evidence for an association of chronic exercise with CRP reduction, but for IL-6 and α-amyloid the findings remain still conflicting. Modification of body composition through body fat reduction was most strongly related to a reduction in inflammatory factors in interventional studies. Increases in adiponectin were observed in exercise interventional studies where participants experienced significant body weight loss. On the contrary, modest changes in both body composition and weight loss by applying exercise for a short period of time failed to modify adiponectin levels, in contrast to the long-term adoption of a moderate-intensity exercise program [59]. Exercise seems to interrupt the metabolic cycle of chronic inflammation, both directly through the production of anti-inflammatory cytokines during each training session and indirectly through the improvement of comorbidities and cardiovascular risk factors by reducing the amount of visceral fat [60,61].

### 3.6. Exercise Targeting on Inhibition of Tumor Cells Survival

Substantial literature has demonstrated the beneficial effects of exercise targeting directly on tumor metabolism, through the inhibition of proliferation and the provision of cancer cell apoptosis. In addition, exercise might prevent the transformation of carcinoma in situ through lowering blood glucose and improving insulin sensitivity [62]. Certain studies in exercising animals and in animals with very low levels of physical activity have shown that specific genes involved in multiplication, aggressive behavior, and cell survival are reduced in the tumor RNA of exercising animals. The exact mechanism remains unknown and may be involved in the development of neoplasms in humans [31]. Exercise can provide the release of tumor suppressors, like programmed cell death protein 4, in a mouse model of estrogen receptor-positive breast cancer. In addition, exercise–exertion dependent catecholamines can diminish breast cancer cell growth through triggering the Hippo tumor suppression metabolic cascade and enhancing the activation of tumor suppression protein p53, induced by exercise sessions. This can lead to tumor growth suppression, as has been evidenced in mouse models of lung and skin cancer [63]. Myocines produced by active muscles like oncostatin M, irisin, and SPARC can directly downregulate cancer growth by impacting cell proliferation, stemness, apoptosis, drug resistance, metabolic remodeling, and the epithelial–mesenchymal tissue transformation of cancer cells [34].

### 3.7. Exercise and Energy Expenditure

The importance of energy balance in carcinogenesis has been known for half a century. Caloric restriction is obviously protective against carcinogenesis and exercise is the other side of the same coin (increased energy expenditure through exercise implementation–reduced caloric intake through diet). However, the physiological and biochemical changes that follow exercise do not take place with caloric restriction [64]. Obesity is a risk factor for many types of cancer. Thus, many researchers link exercise to cancer risk reduction through increased energy expenditure and reduced body fat, while most of them have concluded that exercise is independently linked to a reduction in carcinogenesis risk. Abdominal obesity has also been associated with certain types of cancer, such as bowel, pancreatic, and breast cancer, which has been ascribed to the increased insulin resistance and hyperinsulinemia. Exercise appears to modify these levels and may lead to a reduction in abdominal fat and to supporting muscle mass. The exercising skeletal muscles alter body composition and enhance rest metabolic rate [28,53].

### 3.8. Exercise and Type of Cancer

Regular exercise promotes protection against certain types of cancer, such as breast, colon cancer, and possibly prostate, endometrial, lung, and pancreatic cancer, while for ovarian and testicular cancer there is insufficient evidence to support this relationship [9,65,66]. Exercise is a simple and low-cost non-pharmacological intervention that is of great importance for cancer prevention, also improving the prognosis of cancer patients, promoting prolonged survival. Exercise’s molecular mechanisms against tumor growth, mutation, and the treatments of pharmacological side effects are presented in Table 1.

#### 3.8.1. Exercise and Colon Cancer

The most widely studied cancer in relation to the effect of exercise is colorectal cancer, particularly that located in the colon [10,77,78]. Almost 14% of colorectal tumor malignancies in the population are ascribed to a physically inactive lifestyle [16]. Most studies have observed a 70% reduction in the risk of colon cancer in individuals with higher levels of physical activity. The protective role of physical activity may be related to the incidence of colorectal precancerous polyps. Epidemiological studies have documented that there may be an association of exercise with the risk of developing colonic polyps. In fact, it was observed that those who exercised ≥ 1 h weekly exhibited a lower incidence of colorectal polyps and adenomas compared to those who exercised for <1 h. Moreover, exercise decreased the likelihood of developing polyps in the entire colon, independently of any specific colon location. Additionally, exercise has been found to decrease the whole number of intestinal polyps by 50% and the number of large polyps by 67% [73].

Both occupational and leisure time physical activities have significantly been associated with a reduction in colon cancer risk. The relative risk in cohort studies has been found to be higher compared to case control studies (RR = 0.85 vs. RR= 0.73) [31]. The mean risk factor reduction was found to be 40% to 50%, while the association dose–response was shown to be strong, being increased when exercise was combined with diet and BMI reduction [78]. The potential effects of exercise have been found to include increasing intestinal motility, reducing transit time, reducing fecal bile acids, and improving the immune system (up to two-fold elevation in macrophage activity immediately after high-intensity exercise). Additionally, high physical activity increases the levels of prostaglandins in particular PGF2a, which is inversely related to the prostaglandin PGE2 of the mucosa (having an “oncogenic behavior”) of the colon. The outbreaks of adenomatous polyposis have been associated with Program Cell Death (PD). Exercise may also be associated with Ki-ras oncogene mutations. Individuals with increased PD were less likely to have these mutations compared to non-active individuals [30,31,32]. Long-duration aerobic exercise has also been shown to exert a protective effect against colon cancer, whereas non-association was found between exercise and rectal cancer risk [9].

#### 3.8.2. Exercise and Breast Cancer

Exercise has been shown to reduce the risk of breast cancer by 30–40% in women with increased physical activity and even a dose–response relationship has been found in menopausal women [65,78,79]. The dose–response relationship of exercise has not been found to be linear. In other words, as the dose of exercise increases, the risk factor does not decrease proportionally [79]. No relations have been observed for premenopausal women, while for all women a modest risk reduction from 10% to 20% percent has been reported. Notably, females who met at least five of the World Cancer Research Fund and American Institute for Cancer Research recommendations had a 60% lower risk of breast cancer [80]. Remarkably, for each additional hour of exercise per week, there has been a 6% risk reduction [79]. Moderate aerobic exercise five times a week combined with daily physical activity has reduced risk by 50–75% in postmenopausal women [81]. Aerobic exercise can also be beneficial for both the prevention and therapy of the cardiotoxicity impacts of doxorubicin and trastuzumab, promoting cardiorespiratory capability. In this aspect, the beneficial impact of upper extremity exercise on the controlling of lymphedema in breast carcinoma has been well established, bringing down the myth of restricting the usage of the influenced hand. Sixty percent of breast cancer patients experience body weight increase through simultaneous chemotherapy, which additionally elevates the probability of relapse. Exercise seems to decrease this probability and, specifically, aerobic exercise both during and next to therapy can improve quality of life, decreasing fatigue in breast carcinoma patients [82].

On a molecular basis, exercise stops the metabolic cascade of chronic inflammation by reducing the levels of IL-6 and CRP and inhibiting inflammation progress. Exercising muscles lead to the deceasing of TNFα and α amyloid [51,52,53,54,55,56,57,58,60,61]. Moreover, serum under exercising conditions has been demonstrated to switch off Hippo/Yes-Associated Protein (YAP) signals in breast cancer cells by a mechanism regulated by epinephrine while the blockade of adrenergic signals mitigates the depressant role of exercise serum on both tumor development and cell survival [60,61,63].

#### 3.8.3. Exercise and Endometrial Cancer

Exercise has been related with reduced risk of endometrial cancer incidence. Increased leisure time physical activity has been demonstrated to reduce by about 80% the risk of endometrial cancer. On the contrary, occupational physical activity has been found to exert a non-effect on the decline of endometrial cancer risk [28,78,79]. Exercise can help reduce the risk of death from endometrial cancer by reducing the rates of obesity and by regulating lipoproteins, insulin resistance, and endogenous sex hormone concentrations [46]. It is worth noting that moderate-intensity exercise may have a remarkably positive-association with increasing survival rates, while high-intensity exercise has not [83]. Moreover, exercise has been shown to improve cardiorespiratory fitness, quality of life, and mental health in obese and non-obese women who have experienced endometrial cancer stage I-IIIa [84].

#### 3.8.4. Exercise and Ovarian Cancer

Ovarian cancer is the most common leading cause of death compared to other gynecological cancers. However, women both during and after treatment for ovarian cancer in stages I-IV of the disease are not willing to engage in exercise and do not follow the suggested exercise recommendations [85]. No significant relationship has been observed between the risk of this type of cancer and the effect of exercise or physical activity. The results of the currently available studies are contradictory with some of them supporting a moderate and inverse relation between physical activity and cancer risk, reporting a reduction in the risk of developing invasive ovarian endothelial cancer in women with intense physical activity [65,66]. Exercise has been associated with reduced ovarian cancer risk and improved clinical outcomes. However, the underlying molecular mechanisms remain unknown. Low levels of physical activity have been associated with all histopathological types of epithelial ovarian cancer and higher levels of physical activity seem to reduce the adverse effects of ovarian cancer treatments. Notably, exercise intervention (even at home) has improved quality of life, cardiorespiratory function, and muscle strength, reducing disease and/or disease treatment-related fatigue in women with ovarian cancer. Despite these clinical findings, there is limited research on the molecular mechanisms mediating the effect of exercise on improving ovarian cancer outcomes. Thus, it remains unclear whether physical activity directly or indirectly could affect ovarian cancer incidence and mortality due to the lack of research studying these mechanisms [47].

#### 3.8.5. Exercise and Prostate Cancer

Multiple potential molecular pathways have been suggested to connect exercise and prostate carcinogenesis through mediating the circulating levels of IGF-1, oxidative stress, systemic inflammation, sex hormones, and myokines [48]. Studies investigating the relationship between physical activity and prostate cancer risk have demonstrated controversial results and presented unclear outcomes [30]. However, an overall prostate cancer risk reduction ranging from 5% to 65% has been associated with leisure time physical activity from 10% to 56%, which has been correlated to occupational physical activity. Interestingly, a large Scandinavian study including over one million male participants (73.2% between 60 and 80 years of age, 18.3% > 80 years) has reported an overall reduction in prostate cancer rates of 7–12% for those with a high workload. These results were confirmed by Benke et al. who reported a 17% reduction in prostate cancer risk in men over 60 years of age with long-term occupational physical activity [3,4].

The large main differences in prostate cancer mortality worldwide may be partly ascribed to differences in physical activity evaluation patterns [30]. The association of cancer risk reduction with exercise has not been found to be as strong as it was expected. However, a 54% reduction in metastatic cancer risk has been reported in the high physical activity category of men, with most studies reporting a reduction of about 10–30% [32]. The above findings contradict the conclusion of other studies, where the relative risk was found to be independent of exercise intensity, while in others studies the duration and frequency of exercise has been associated with cancer incidence. One possible molecular mechanism of exercise involved in risk reduction appears to be the increased testosterone binding by the musculoskeletal system because of chronic and high-intensity exercise. Resistance exercise has also led to a rise in testosterone that was offset by an increase in androgen receptors in exercising muscles [9,30,32]. In prostate cancer, 3 h per week of vigorous activity (jogging, cycling, swimming, tennis, and weight training) has led to a 70% reduction in the risk of high-grade, progressive, or fatal prostate cancer [13]. The magnitude of the effect of exercise on a wide variety of bodily functions appears to be extensive. Some of the beneficial metabolic effects may be influenced by the anti-inflammatory response, immune system, cognitive function, hematopoietic system, estrogen production, IGF-1, apoptosis, deoxyribonucleic acid damage, and genome diversion (gene expression and mutation) [86].

#### 3.8.6. Exercise and Pancreatic Cancer

Pancreatic cancer is one of the most aggressive and early metastatic tumor malignancies with poor prognosis worldwide. It is expected to have a double incidence in 2030. Physical inactivity appears to increase the risk of pancreatic cancer. Total physical activity in subjects with BMI < 25 kg/m^2^ has not been related with the risk factor. In contrast, exercise training, through improvement of glucose tolerance and weight loss in overweight and obese people, has been found to reduce pancreatic cancer risk. Vigorous physical activity has not been associated with cancer risk, while moderate physical activity has inversely been associated with cancer risk. Individuals, both male and female, who walked more than four hours per week have showed a 54% lower risk of pancreatic cancer compared to those who spent less than 20 min walking weekly, with a significant reduction in relative risk (RR = 0.45) [41]. 

Investigations of the exercise mechanisms leading to pancreatic cancer risk induction and tumor suppression have also been limited. Scientific evidence has revealed contradictory results and particularly few studies have supported that exercise can reduce tumor growth when chemotherapy agents have not been delivered. Recent research has highlighted that not only the absence of chemotherapy medicine but the volume and intensity of exercise training may be associated with tumor growth, while others have provided positive response to cancer suppression and growth reduction during chemotherapy implementation [67]. Both the endurance and resistance exercise of moderate intensity seem to enhance the psychological status and improve the functional capacity of the affected people. High-intensity interval training has also showed a negative correlation with pancreatic tumor growth in patients who underwent medical treatment [87]. Aerobic exercise combined with chemotherapy has exhibited a greater effect on tumor growth reduction through the activation of calcineurin-NFAT-TSP1 signaling in endothelial cells, exerting a key role in exercise-induced shear stress mediated tumor vessel remodeling [67].

#### 3.8.7. Exercise and Lung Cancer

Lung cancer is the underlying cause of almost all cancer-related deaths worldwide and non-small-cell lung cancer (NSCLC) represents 85% of all lung tumor malignancies, presenting survival rates less than 5 years. Three main histological forms of NSCLC have been described as large-cell carcinoma (10–15%), adenocarcinoma (40%), and squamous cell carcinoma (25–30%). Adenocarcinomas represent the majority of non-small lung cancer cases and there is evidence that its incidence rates are increasing worldwide compared to other lung carcinomas [88]. Elevated cell proliferation, decreased apoptosis, and prolonged survival have been considered as the main features of cancer cells, which are induced by the modulation of key signaling molecules/single pathways and thus may activate a cascade of biological reactions. The above cellular mechanisms are induced or regulated by the anti-cancer molecular mechanisms of exercise. 

Physical activity has positive effects not only in the prevention of lung cancer, but also in the treatment and prognosis. Exercise has been shown to reduce chronic inflammation and oxidative stress in both normal and pathophysiological lung conditions, like lung carcinoma and/or cystic fibrosis, promoting molecular mechanisms that can activate anti-inflammatory mediators. Moreover, exercise has been shown to influence lung functionality and decrease the probability of infections and pulmonary disorders. There is currently considerable evidence that diverse mechanisms may be involved in the relationship between cancer and physical activity through the regulation of chronic inflammation and the regulation of different substances that can act as part of metabolic dysregulation such as insulin, glucose, and sex hormones. In addition, physical activity seems to exert an impact on oxidative stress and immune function by modifying some critical molecular mechanisms related to the tumor microenvironment such as angiogenesis, cell proliferation, and apoptosis [89].

Elevated cell proliferation, decreased apoptosis, and prolonged survival are the main features of cancer cells, which are induced by the modulation of key signaling molecules/single pathways and by activating a cascade of reactions. Phosphatidylinositol-3- kinase (PI3K)-Akt and the kinase that is regulated by the Ras-extracellular signaling (Erk1/2) have been stimulated by attaching growth factor to cell surface receptors, resulting in elevated cell proliferation and surveillance [68]. The activated Akt has triggered the following mechanisms of rapamycin (mTOR), which in response can activate ribosomal kinase S6 (p70 S6K), leading to the augmentation of cell proliferation and protein synthesis [69]. Akt has also induced phosphorylation and degeneration of the pro-apoptotic Bad protein, which has resulted in both suppression of apoptosis and increased survival, being involved in cancer cell therapy resistance [70]. On the other hand, the Ras signaling molecule has been shown to be mutated/activated in cancer, including NSCLC, leading to the subsequent activation of Erk1/2 and resulting in cell proliferation and resistance to chemotherapy and radiation [71]. Therefore, identifying strategies that can restrict the Akt and Ras-Erk1/2 signaling cascades may be an effective therapeutic approach for the treatment of lung cancer.

Regular exercise is generally related to overall health benefits and a decreased risk of many types of cancer. Although the relation between lung cancer and exercise has not comprehensively been investigated, exercise at a moderate intensity (>4.5 METs) and a frequency of more than four times a week has been found to be effective in lung cancer risk reduction compared to people following exercise training at lower intensity and frequency [65]. Hence, adaption of exercise over a long period of life may exert a positive effect on reducing the risk of lung cancer. The reduction differs between genders, with women being at a greater advantage than men, and it is greater for people with high physical activity [66]. Interestingly, an inverse association of vigorous leisure time physical activity and lung cancer risk has been found, while strenuous severe occupational physical activity has been shown to exert a larger effect on the reduction of lung cancer risk [28]. Thus, lung cancer cases in men and women with sedentary lifestyles could have been prevented by including regular exercise in their daily lives [66].

#### 3.8.8. Exercise and Gastric Cancer

Nowadays, the incidence rates of gastric cancers tend to decline. However, it remains the most common cause of death among men compared to women. More than one million new cases of stomach cancer are detected worldwide every year. Stomach cancer is also one of the most behaviorally influenced cancers and is therefore preventable [90]. Regular exercise training has possibly affected health-related quality of life, contributing to gastric cancer risk reduction. Nevertheless, research findings are scarce and contradictory. Studies have reported exercise side effects in this subgroup of oncological patients such as nausea, vomiting, gastrointestinal breeding, reflux, depression, fatigue, and lack of motivation. On the other hand, the indirect outcomes of sustained regular aerobic exercise have been demonstrated [91], which may result in muscle mass and strength elevation, as well as the improvement of functional capacity and psychological status with a regular individualized exercise program adapted to patients’ needs and clinical conditions [71]. However, it has been elucidated that an aerobic and resistance exercise program of 30 min twice a week has resulted in the reduction of plasma 3-hydroxy-kynurenine (HK), a major compound that relates to depression, and of the neurotoxic metabolites of gastric cancer patients [92]. Nevertheless, most of the published studies which have investigated the effect of exercise on gastric cancer have been focused on the pre- and/or post-operative period of the affected patients. Adoption of an exercise program based on Frequency–Intensity–Time–Type (FITT) principles and the objective perception of exercise fatigue has improved the functional capacity and cardiorespiratory function of esophageal and gastric cancer patients who underwent neoadjuvant treatment preoperatively [92]. Extensive investigation is further required in order to detect the exact dominating molecular mechanisms through which exercise may elicit benefits in gastric cancer patients.

#### 3.8.9. Exercise and Skin Cancer

The main types of skin cancer include melanoma, basal cell carcinoma, cutaneous squamous cell carcinoma, and Merkel cell carcinoma. To the best of our knowledge there is limited literature on the examination of the association between exercise and skin cancer. The association of exercise with the likelihood of cutaneous squamous cell carcinoma (SCC) remains still unknown and is problematic to examine because of the confounding exposure to sunlight [74]. There is no considerable association of recreational activity tools with SCC next to adjusting for possible confounding factors, containing sun exposure indices. In men, the observed risk motif has proposed an enhanced risk with elevating total hours of leisure-time activity. Concerning women, the higher amount of occupational activity (standing and manual work vs. sedentary work) has been associated with a decreased prevalence of developing SCC tumors (*p* = 0.03) [74].

However, exercise appears to have an important role for confronting melanoma by modifying energy imbalance and by promoting and upregulating ceramides. Ceramides are bioactive molecules, which can promote the proliferation and apoptosis in cancer cells. It has been highlighted that aerobic exercise may activate and also increase ceramides, which in turn could act as apoptotic agents of tumor cells [93]. Few studies have also examined genes induced by exercise and the prognosis of skin melanoma and particularly by providing the expression of tumor suppression genes [74].

#### 3.8.10. Exercise and Liver Cancer

Exercise seems to have a protective role against liver cancer risk and disease progression. However, this relationship has not been defined because of the lack of interventional studies in humans. Notably, lower rates of mortality between active and less active liver cancer patients have been observed [94]. Particularly, regular exercise has been shown to reduce the risk of hepatocellular cancer. Exercise seems to activate adenosine monophosphate protein kinase (AMPK), phosphorylating the regulatory protein related to the mammalian target of rapamycin (raptor), which in turn may lead to the inhibition of the mammalian target of rapamycin (mTOR) complex 1 (mTORC1). The mTOR has been considered to act as a crucial regulator of cell proliferation, tumor growth, and survival in response to molecular growth factors and nutritional status. AMPK has been shown to function as an energy sensor in cells, exerting a dominant impact in connecting metabolism and cancer [75]. Nonetheless, molecular mechanisms by which exercise can inhibit cancer cell growth and metastasis are still unclear. Substantial research has also demonstrated that regular exercise may improve the quality of life of liver cancer inpatients after diagnosis without deteriorating liver function [95] and has also reduced the extent of the tumor [96]. Moreover, regular physical activity has been shown to exert a profound effect on the prognostic transcriptional signature by downregulating most of the genes involved in poor survival and upregulating the most genes related to optimal prognosis [76].

## 4. Discussion

Undoubtedly, exercise can exert a protective effect against cancer. It could be mentioned that it may act as a preventive “medication” that should be applied throughout the lifetime. The great benefits of exercise for the general population are pointing at the implementation of similar physical activity practices in the field of cancer education and prevention. The American Cancer Society, in cooperation with the American Heart Association and the World Health Organization, has effectively promoted recommendations for adopting a more active lifestyle by recommending for adults 150 min per week of moderate-intensity exercise or 75 min per week of high-intensity exercise or a combination of these, while limiting sedentary behavior. However, these levels of physical activity appeared to be associated with a reduction in the incidence of cancer morbidity and mortality. The optimal frequency, intensity, duration, and type of exercise required to reduce cancer risk remain unclear. It has been speculated that 300 min of moderate-intensity activity per week or 150 min of vigorous activity per week, continuous session or in separate sessions of 20 to 30 min each, may exert an additional protection against cancer either directly or indirectly by preventing or even decreasing body weight gain and obesity.

However, the potential interrelationship between exercise and cancer remains still unclear. Both the heterogeneity of cancer and the heterogeneity of the population under study at any given time as well as the different methods of assessment of the intensity, duration, frequency, dose, and timing of exercise in the lifespan, together with the biochemical and molecular basis of the interactions between exercise and carcinogenesis, are limiting factors in almost all the studies. Different types and intensities or progression of exercise may lead to different responses and adaptations by the immune and endocrine systems with the involvement and modification of many hormones. Moreover, the kind of exercise may play a distinct role and future studies should take the above under consideration as there is not conclusive evidence in this issue so far. Collectively, further investigations which may better predict the exercise–cancer interrelationship by highlighting the relationship among exercise biochemistry, physiology, and cancer cell metabolism through intervention studies are strongly recommended. It should be also kept in mind that angiogenesis is a complex and multifaceted process with different steps which can be influenced by exercise; however, there is currently not enough data on this issue and thus future studies should be performed on this issue.

However, a large body of literature demonstrates the effect of exercise on cancer patients’ functional capacity and does not reveal the molecular mechanisms supported by exercise biochemistry in combination with tumor metabolism and microenvironment. Obviously, such studies could be performed by using animal subjects. On the other hand, it seems to be a research gap in implementing exercise in pediatric and young adults’ oncology treatment, too. Additionally, only a few interventional studies have considered anti-cancer medication and the relationship with exercise modalities in humans, including a small sample of participants which is a restriction to generalize results. 

## 5. Conclusions

Currently, several molecular mechanisms by which exercise can decrease cancer risk and/or progression have been found. The most important and well-defined of them include the metabolic profile of hormones, the systemic inflammation reduction, the insulin sensitivity increase, the antioxidant capacity augmentation, the boost to the immune system, and the direct effect on the tumor. The currently available data provide substantial evidence that the effect of exercise may predict a stronger association with cancer and could supplementarily be introduced in the cancer clinical practice to improve disease progression and prognosis. Future studies should apply direct measurements of metabolic energy equivalence or maximal or peak oxygen intake or heart rate in combination with new biochemical markers and not only the assessment of the level of exercise by self-reported methods. Future research should limit or control confounding factors such as heredity, lifestyle factors, comorbidities, medication, and type of occupation in relation to exposure to environmental irritants in the study population.

## Figures and Tables

**Table 1:** The most well-established anti-cancer molecular mechanisms of exercise.

Type of Cancer	Exercise’s Anti-Cancer Molecular Mechanisms	References
Breast cancer	↓ IGF-1, TNFα, IL-6, amyloid-α, CRP ↓ CRP stop metabolic cascade of chronic inflammation, Switch of Hippo/Yes-Associated Protein signaling	[51,52,53,54,55,56,57,58,60,61,63]
Ovarian cancer	↓ Cardiotoxicity by chemotherapy treatment	[65,66]
Endometrial cancer	↑ The rates of obesity Regulates lipoproteins, insulin resistance, and endogenous sex hormone concentrations Lack of studies on molecular mechanisms	[46,47]
Prostate cancer	↑ Testosterone binding by the musculoskeletal system as a result of chronic and high-intensity exercise Resistance exercise increases testosterone that is offset by an increase in androgen receptors in exercising muscles ↑ Deoxyribonucleic acid damage, and genome diversion ↓ IGF-1 levels	[9,13,48]
Pancreatic cancer	Aerobic exercise combined with chemotherapy increases calcineurin- NFAT-TSP1 signaling and remodels tumor vessels	[33,41,67]
Lung cancer	Modulation of Ras-Erk1/2 signaling cascades ↑ Restriction of the Akt and Ras-Erk1/2 signaling cascades may be an effective therapeutic approach for the treatment of lung cancer under consideration	[28,66,68,69,70,71]
Gastric cancer	Aerobic + resistance exercise, 2t/week × 30′ decrease plasma neurotoximetabolites such as 3-hydroxykynurenine	[72]
Colorectal cancer	↑ Macrophage activity after high-intensity exercise ↓ 50% polyps in the entire colon Increasing the number of NK cells’ function and cytotoxicity High physical activity levels increase the levels of prostaglandins (PGF2a,) which is inversely related to the prostaglandin PGE2 of the mucosa; exercise may reduce Ki-ras oncogene mutations. ↑ Program cell death	[30,31,32,73]
Skin cancer	Higher levels of ceramides increase apoptotic agents of tumor cells	[74]
Liver cancer	Activates adenosine monophosphate protein kinase Phosphorylates mammalian target of rapamycin (MTOR),-complex 1 Downregulates genes involved in poor survival	[75,76]

## Data Availability

The data of the study are available upon request to the corresponding author.
